# Proteome-wide characterization of signalling interactions in the hippocampal CA4/DG subfield of patients with Alzheimer’s disease

**DOI:** 10.1038/srep11138

**Published:** 2015-06-10

**Authors:** Jae Ho Kim, Julien Franck, Taewook Kang, Helmut Heinsen, Rivka Ravid, Isidro Ferrer, Mi Hee Cheon, Joo-Yong Lee, Jong Shin Yoo, Harry W Steinbusch, Michel Salzet, Isabelle Fournier, Young Mok Park

**Affiliations:** 1 Center for Cognition and Sociality, Institute for Basic Science, Daejeon 305-811, Republic of Korea; 2 Mass Spectrometry Research Center, Korea Basic Science Institute, 804-1 Yangcheong-ri, Ochang-eup, Cheongwon-gun, Chungbuk 363-883, Republic of Korea; 3 Inserm U-1192, Laboratoire de Protéomique, Réponse Inflammatoire, Spectrométrie de Masse (PRISM), Université de Lille 1, Cité Scientifique, 59655 Villeneuve D’Ascq, France; 4 Graduate School of Analytical Science and Technology, Chungnam National University, Daejeon 305-764, Republic of Korea; 5 Department of Psychiatry, Morphological Brain Research Unit, University of Würzburg, Würzburg, Germany; 6 Brain Bank Consultants, Amsterdam, The Netherlands; 7 Institut de Neuropatologia, Servei Anatomia Patologica, IDIBELL-Hospital Universitari de Bellvitge, Universitat de Barcelona, Spain; 8 School for Mental Health and Neuroscience, Department of Translational Neuroscience, Maastricht University, Maastricht, The Netherlands

## Abstract

Alzheimer’s disease (AD) is the most common form of dementia; however, mechanisms and biomarkers remain unclear. Here, we examined hippocampal CA4 and dentate gyrus subfields, which are less studied in the context of AD pathology, in post-mortem AD and control tissue to identify possible biomarkers. We performed mass spectrometry-based proteomic analysis combined with label-free quantification for identification of differentially expressed proteins. We identified 4,328 proteins, of which 113 showed more than 2-fold higher or lower expression in AD hippocampi than in control tissues. Five proteins were identified as putative AD biomarkers (MDH2, PCLO, TRRAP, YWHAZ, and MUC19 isoform 5) and were cross-validated by immunoblotting, selected reaction monitoring, and MALDI imaging. We also used a bioinformatics approach to examine upstream signalling interactions of the 113 regulated proteins. Five upstream signalling (IGF1, BDNF, ZAP70, MYC, and cyclosporin A) factors showed novel interactions in AD hippocampi. Taken together, these results demonstrate a novel platform that may provide new strategies for the early detection of AD and thus its diagnosis.

Alzheimer’s disease (AD) is one of the most common neurodegenerative disorders, and its prevalence is estimated to increase two-fold in industrialized countries and by four-fold in developing countries over the next 20 years^1^. AD is characterized by a loss in connectivity in several brain fields in particular including the hippocampus. For instance, the density of synapses was significantly decreased in the outer molecular layer of the hippocampal dentate gyrus in AD^2^. Notably, the hippocampus plays a key role in the neurodegenerative events occurring in AD, since changes in this field are closely associated with memory loss and cognitive impairment^3^. Despite recent progress in understanding the mechanism of AD, our knowledge of the molecular details regarding this disease is still incomplete.

Many previous studies of AD have focused on the CA1 and CA3 fields of the hippocampus in various species^4^,^5^. By contrast, our laboratory has focused on these hippocampal fields less studied in the context of AD pathology. Notably, the DG plays a major role in age-related memory impairment but relatively little is known about the molecular mechanisms responsible for this memory loss^6^. Furthermore, pathological changes in the DG, including granule cell loss and mossy fibre sprouting, are closely associated with status epilepticus^7^. Moreover, potential proteome-wide changes in the CA4 field of AD or their biological function remain unknown. In general, the lack of research on the DG and CA4 fields could in part be due to the lack of a clear distinction between the DG and CA4 in terms of their function and role in AD. This lack of clarity is why we specifically selected these fields from hippocampal tissues of AD patients and compared them to control samples.

Key initiating factors of AD include the accumulation of intraneuronal neurofibrillary tangles (NFTs) and the appearance of extracellular β-amyloid deposits^8^,^9^. One of the main histological findings in the brains of AD patients is the intracellular accumulation of NFTs^10^,^11^. Other histopathological features include the accumulation of extracellular senile plaques comprised of aggregated β-amyloid peptides. However, the molecular mechanisms associated with AD pathogenesis are not entirely understood. Moreover, some areas of the hippocampus are vulnerable to specific degenerative processes depending on their subfield and may differentially express proteins in specific stages of the disease. Intraneuronal neurofibrillary changes exhibit a characteristic pattern according to pathological stage of AD^12^. Therefore, in the current study, we focused on relatively early AD pathology, when accumulation of NFTs and neuropil threads are first detected in the CA4/DG^12^ to elucidate mechanisms involved in the onset and development of AD. To accomplish this, we used hippocampal specimens staged at AD Braak -IV and AD Braak -V. Using the differentially-expressed proteins identified from the CA4/DG field, we also examined protein-protein interaction networks associated with the mechanisms of AD through a bioinformatics approach. An examination of the signalling interaction networks associated with AD can provide insights into the molecular events mediating AD pathology. In addition, it can provide identifying potential new diagnostic or prognostic biomarkers.

Most previous studies that aimed to identify biomarkers for AD diagnosis have predominantly employed positron emission tomography (PET) and magnetic resonance imaging (MRI) approaches^13^. However, new tools have now been developed which are highly more sensitive and specific and are able to detect biomarkers relevant to disease progression and the signalling pathways underlying AD. Thus, in the current study, we combined mass spectrometry-based proteomic analysis with label-free quantification for identification and quantification of proteins from AD and control samples. We dissected the CA4 and DG fields from human hippocampal tissues and used one-dimensional liquid chromatography-tandem mass spectrometry (LC-MS/MS) with subsequent database searches to identify peptides. Using this methodology, we identified previously unknown pathological mechanisms of AD. Additionally, the use of multiple high-throughput technologies enabled the detection of novel proteins and cellular signalling pathways associated with AD pathology. Among the proteins identified, we targeted five regulated proteins (MDH2, PCLO, TRRAP, YWHAZ, and MUC19 isoform 5) and cross-validated them by using western blot analyses, selective reaction monitoring (SRM), and matrix-assisted laser desorption/ionization (MALDI) mass spectrometry imaging (MSI). Thus, we combined leading proteomic technologies for use as a diagnostic tool for the identification novel AD biomarkers and candidate drug targets. Our results provide valuable clinical information and can be utilized as a novel platform for the early diagnosis of AD.

## Results

### AD biomarker identification

**Figure 1:**
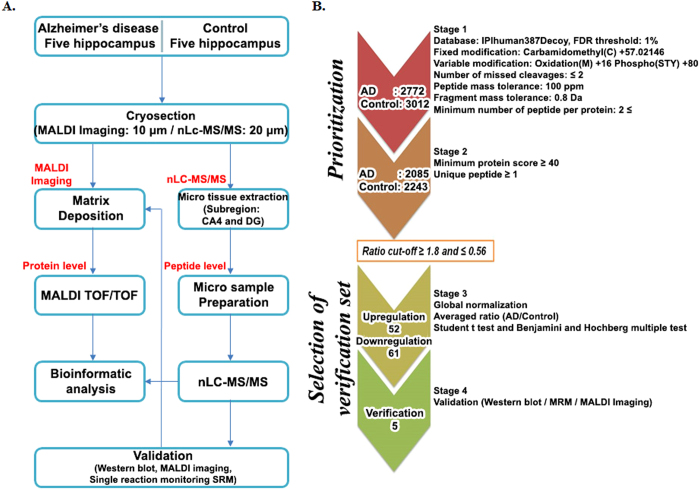
Strategy for identifying putative biomarkers of AD. (**A**) Experimental scheme for analysis of regulated proteins from post-mortem AD tissues using mass spectrometry-based proteomic analysis with label-free quantification. (**B**) Protein prioritization scheme for the selection of candidate proteins for verification.

**Figure 2:**
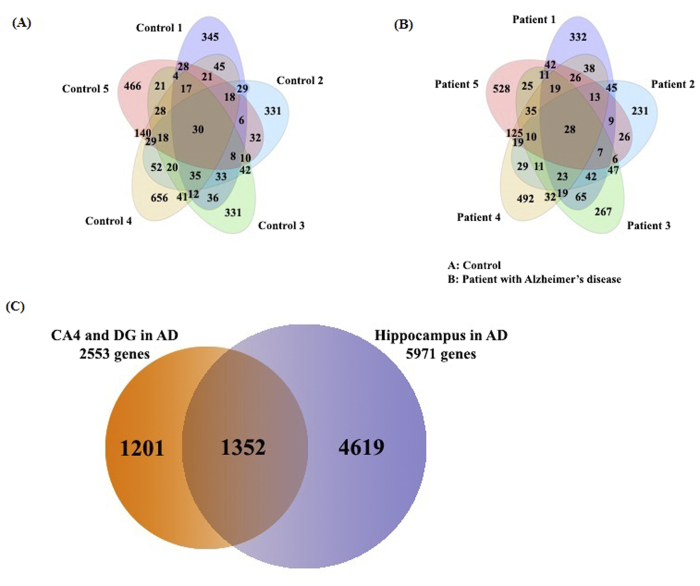
Venn diagrams summarizing proteins identified in human hippocampal tissue obtained from Alzheimer’s disease and control patients. (**A**) Overlap of proteins identified in all five control hippocampi, (**B**) Overlap of proteins identified in all five AD hippocampi, (**C**) Overlap of proteins identified by comparison between the CA4 and DG subfields and the entire hippocampus.

To select meaningful proteins from total identified proteins in AD and control hippocampi, we performed a systematic data-mining strategy using an in-house protocol that involved four stages (Fig. 1). A total of 5,784 proteins from AD and control hippocampal tissues were extensively profiled by high-resolution nanoLC/MS throughout stage 1 (Fig. 1B). We identified 4,328 unique proteins from five AD hippocampus samples (2,085) and five control samples (2,243) (Fig. 2A,B) using a minimum of one unique peptide and a protein FDR of ≤1%. Thereafter, we adopted a ratio cut-off of ≥1.8 and ≤0.56 and obtained 113 proteins (52 upregulated proteins and 61 downregulated proteins; stage 3) (Table 1) for the refinedverification set; stage 4 (Fig. 1B). Recently, our institution^14^ identified 5,971 proteins in AD hippocampal tissues by off-gel fractionation, and profiled these proteins by using a high-resolution mass spectrometer with collision-induced dissociation and electron transfer dissociation. Fig. 2C shows the number of identified proteins in the CA4 and DG subfields and the entire hippocampus.

**Figure 3:**
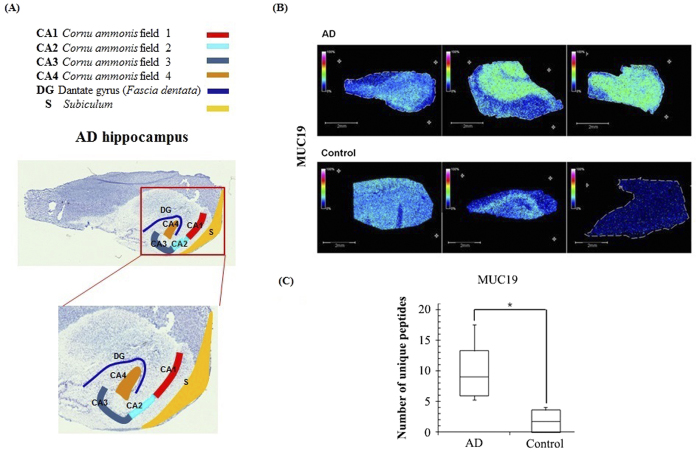
(**A)** Annotated optical image of a hippocampal section obtained from an Alzheimer’s disease patient stained with toluidine blue; Horn of Ammon (CA), dentate gryus (DG), and subiculum (S). (**B**) MUC19 isoform 5 is highly expressed in AD hippocampal tissues. MALDI imaging mass spectrometry (MSI) data (MUC19; m/z 11,135 ± 0.05%). (**C**) Spectral counting data for MUC19.

For our experiments, we selected only five hippocampi per group from the total specimens (Supplementary Information 1) since our criteria discriminated against each subfield of the hippocampus. For this purpose, we first investigated hippocampal histology to identify the individual subfields (Fig. 3A) and coupled our observations to MALDI MSI.

### Validation of proteomic profiling data

**Figure 4:**
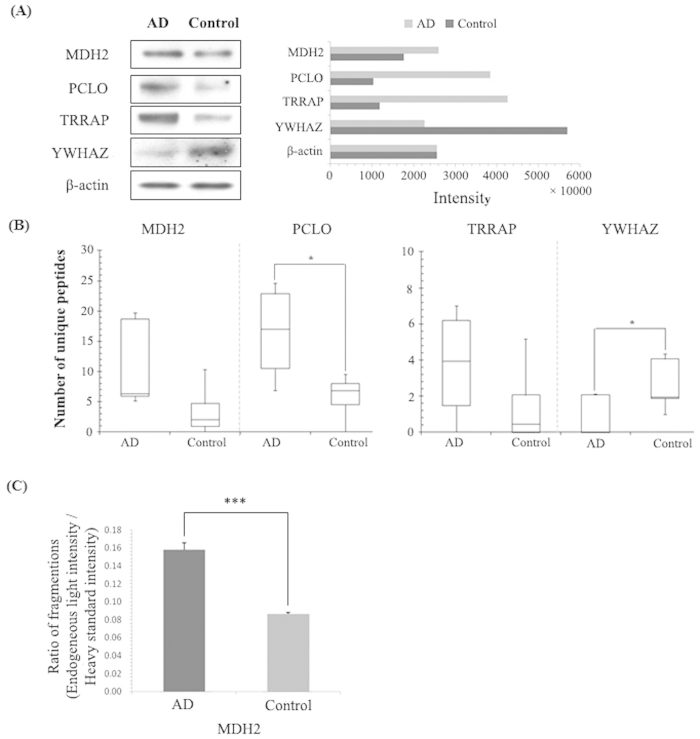
Validation of proteins identified by nanoLC coupled with ESI ion-trap MS. (**A**) Western blot analysis of MDH2, PCLO, TRRAP, and YWHAZ. (**B**) Spectral counting of PCLO, TRRAP, and YWHAZ. (**C**) Comparison of individual protein amounts with the fragmentation ratio (endogenous light peak area/heavy standard peak area) in AD and control tissues as assessed by SRM.

To the best of our knowledge, no previous studies have linked MUC19 isoform 5 with AD until now. Since there are no commercially available human MUC19 isoform 5 antibodies and the sequence of the MUC19 oligomeric protein (isoform 5) is slightly different from the full sequence of Muc19 protein (Supplementary Information 2). We used MALDI MSI and spectral counting to confirm that MUC19 isoform 5 was highly expressed specifically in AD hippocampal tissues in contrary to controls (Fig. 3B, C). We next performed western blot analyses to confirm the expression levels of four proteins (MDH2, PCLO, TRRAP, and YWHAZ, with β-actin as control) identified in the CA4 and DG field of AD hippocampal tissues. Notably, higher levels of MDH2, PCLO, and TRRAP were observed in AD tissue than in control hippocampal tissue (Fig. 4A); however, lower levels of YWHAZ were observed in control tissue. Furthermore, the western blot data for these proteins correlated well with the spectral counting data (Fig. 4B). We also confirmed expression of MDH2 using SRM. As shown in Fig. 4C, MDH2 expression was approximately two-fold higher in AD hippocampi than in control tissue.

**Figure 5:**
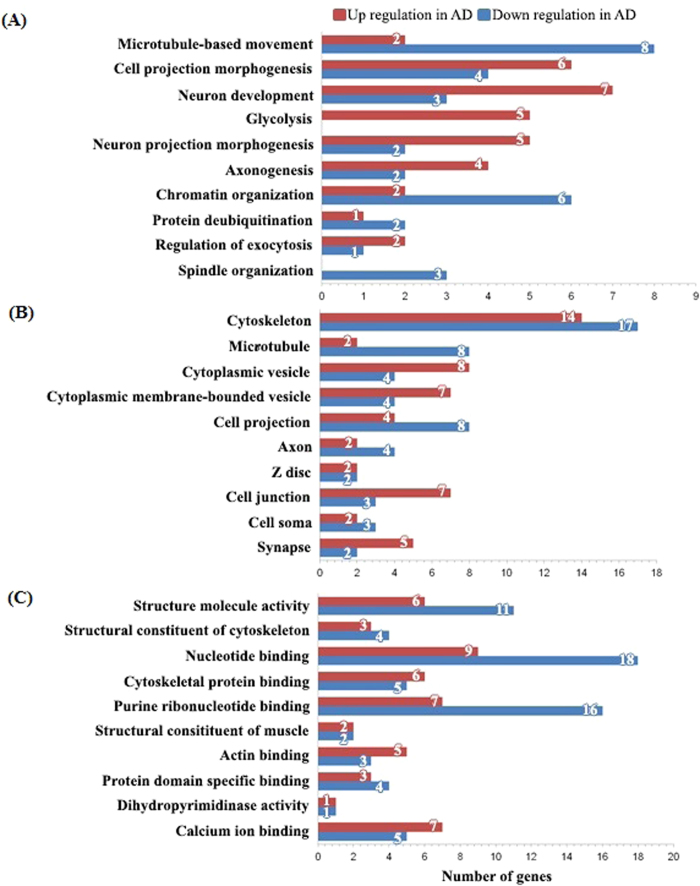
Analysis of regulated proteins using DAVID functional Gene Ontology. (**A**) Biological processes. (**B**) Cellular components. (**C**) Molecular functions (EASE score > 1.3, p < 0.05).

We selected proteins to investigate according to differences in their potential function (Fig. 5A). These unique classifications were composed of several categories including microtubule-based movement, cell-projection morphogenesis, neuronal development, glycolysis, neuronal-projection morphogenesis, and others as listed. Interestingly, proteins that were classified into the glycolysis category (PKM2, PGAM1, ENO3, PGK1, and MDH2) were found to be upregulated in AD tissue. By contrast, proteins classified as being involved in microtubule-based movement and chromatin organization tended to be downregulated in AD tissue (Fig. 5A). A large proportion of the cellular components identified were cytoskeletal proteins (Fig. 5B). In AD tissue, proteins classified as nucleotide binding and purine ribonucleotide binding proteins tended to be downregulated rather than upregulated by more than two-fold (Fig. 5C).

### Upstream signalling interactions of proteins differentially expressed in AD patients

**Figure 6:**
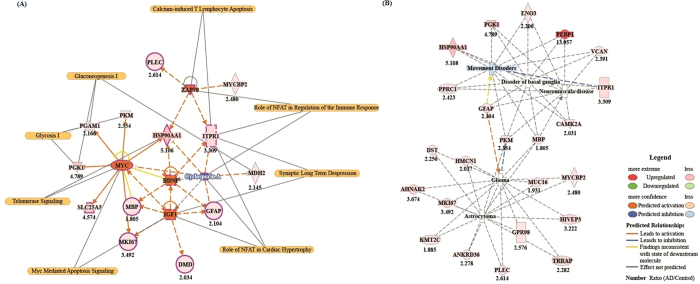
Features of proteomic signalling interactions. (**A**) Signalling network for the relationship between the activation and/or inhibition of upstream regulators with downstream regulated proteins (**B**) Interaction network for canonical neurological disease pathways including proteins that were up/downregulated in AD tissue.

We used Ingenuity Pathway Analysis (IPA) to investigate the potential signalling interactions of the 113 proteins that were expressed more than 2-fold higher or lower in AD hippocampi than in control tissues. We determined the five most significantly annotated upstream regulators by using z-scores and p values (Table 2). These included two growth factors (IGF1, insulin-like growth factor 1; and BDNF, brain-derived neurotrophic factor), one kinase (ZAP-70 tyrosine-protein kinase), one transcriptional regulator (Myc proto-oncogene protein), and one biological drug (cyclosporin A). These factors markedly increased or decreased z-scores. We identified a signalling network representative of the relationship between the activation and/or inhibition of upstream regulators with downstream regulated proteins (Fig. 6A). Through these tools, we also determined a protein interaction network for the potential role of our identified proteins in various neurological disorders (Fig. 6B).

## Discussion

We employed nanoLC coupled with ESI-ion trap MS. Since nanoLC-based MS has the advantages of high spectral resolution and the ability to be used with a shotgun strategy, it can be utilized for identifying a large number of proteins in complex protein mixtures.

Using this approach, we identified 113 candidate molecules in tissue from patients with AD. Among these proteins, approximately 75% had a previously unknown relationship with AD; however, 60% of the 113 proteins had previously been reported to be associated directly or indirectly with the brain. To narrow down our investigation, we selected proteins that had previously been identified in neurological disorders, and validated our proteomic targets by employing western blot analysis and SRM, and MALDI MSI. SRM is particularly useful for validating candidate biomarker that require isotopically labelled standard but have no available antibodies^15^. Moreover, MALDI MSI has emerged as a powerful tool for molecular imaging of thin tissue sections and can provide information about the spatial distribution of proteins in tissue^16^,^17^. The AD-associated proteins that we chose to further investigate included PCLO, which is associated with major depressive disorder (MDD); MDH2, which has been shown to regulate oxidative stress; and TRRAP, which plays an important role, together with Fe65, in AD.

A recent genome-wide association study on MDD identified a specific relationship with a non-synonymous polymorphism (rs2522833) of a gene encoding the presynaptic protein, piccolo (PCLO)^18^, which is localized to nerve terminals of both excitatory and inhibitory synapses in the CNS and influences Ca^2+^-dependent monoamine neurotransmitter release^19^. Bassoon and PCLO are critical regulators of presynaptic ubiquitination and degradation^20^. Surprisingly, in the current study we found that PCLO levels were higher and ubiquitin carboxyl-terminal hydrolase isozyme L1 (UCHL1) levels were lower in the hippocampus of AD in contrary to controls (Fig. 4A). UCHL1 is related to mono-ubiquitin recycling and thereby, sustaining protein degradation by the ubiquitin-proteasome system (UPS). In addition, the down-regulation of UCHL1 in AD is determined by induction of the NF-kB pathway and an Aβ mediated up-regulation of BACE1, by interfering with its lysosomal degradation^21^. Our data suggest that PCLO and UCHL1 could be potential targets for new treatment development.

There were a number of protein changes that were not unexpected between AD and control tissue. One such protein, MDH, is involved in oxidative stress which is a process well known as one of the major causes of AD pathology^22^. During oxidative stress, MDH activity and MDH2 mRNA expression increased by 19% and 22%, respectively, in a mouse hippocampal cell line (HT22)^23^. According to our western blot data (Fig. 4A), MDH2 was more highly expressed in AD hippocampal tissues than in controls. SRM verified that MDH2 expression was approximately two-fold greater in AD tissue (Fig. 4C), and this difference between tissues was also consistently observed with spectral counting.

Another protein of interest, TRRAP, is a well-known cofactor of histone acetyltransferase, which binds to Tip60 to repair double-stranded DNA breaks^24^. TRRAP also plays a key role in the regulation of Fe65, which is a binding partner of the β-amyloid precursor protein, APP, to activate histone H4 acetylation at DNA strand breaks^25^. Therefore, it was not surprising that TRRAP was more highly expressed in AD hippocampi than in controls (Fig. 4A).

The 14-3-3 zeta protein isoform 1 (YWHAZ) is associated with tau phosphorylation in NFTs, which is a characteristic neurohistological marker of AD^26^,^27^. In AD hippocampal tissues, low levels of YWHAZ have been previously reported^27^. Our current data, including the spectral counting and western blotting data, is consistent with this previous report in that we observed lower YWHAZ expression in AD hippocampal tissue than in control tissue (Fig. 4A).

In contrast to the function of YWHAZ, UCHL1 was previously reported^28^ to play an important role in preventing neurofibrillary tangle formation by de-ubiquitination of phosphorylated tau. Interestingly, UCHL1 expression was downregulated in patients with AD (Table 1) compared with that of the controls. We propose that the low levels of UCHL1 expression may have affected AD patients’ ability to prevent NFT formation.

Another protein involved in AD pathology, ITPR1 (inositol 1,4,5-trisphosphate receptor, type 1), is well known as an important protein involved the regulation of Ca^2+^ homeostasis and apoptosis in AD. ITPR1 is localized to the endoplasmic reticulum and interacts with β-amyloid peptide; it is possible that this interaction could influence intracellular calcium homeostasis^29^, induce inactivate eNOS, and ultimately lead to apoptosis. Our data indicated that ITPR1 was approximately three-fold higher in the AD hippocampus than in control tissues.

Levels of dihydropyrimidinase-related protein 2 (DPYSL2; CRMP2), E3 ubiquitin-protein ligase (HUWE1), and sodium/potassium-transporting ATPase subunit alpha-3 (ATP1A3) were significantly lower in AD samples compared with controls. DPYSL2 has been implicated in neuronal growth, polarity, and guidance as well as in AD^30^. Further, chromosomal microduplication or mutation of HUWE1 has been associated with mental retardation^31^. ATP1A3 is known to be a Na+/K+ transporter important in maintaining Na+/K+ gradients in neurons^32^.

The neuron navigator protein family is also of interest. According to our results, neuron navigator 1 was upregulated whereas neuron navigator 2 and 3 were downregulated in AD tissue (Table 1). These proteins are regulators of axon guidance and serve as targets for miRNA. Neuron navigator 1 is a novel microtubule-associated protein, which is involved in neuronal migration^33^, while neuron navigator 2 plays a key role in axonal elongation and formation. It provides stability to growing neurites^34^. Shioya *et al*.^35^ recently reported that a lack of correlation between the mRNA and protein levels of neuron navigator 3. Interestingly, a recent study showed that neuron navigator 3 is involved in tumour suppression^36^.

Notably, IGF1 modulates the hyperphosphorylation of tau through activation of kinases^37^,^38^,^39^,^40^. The cited study^39^ reported that increased IGF1 levels in middle age are associated with a high risk of AD at an older age. In addition, hyper-activation of IGF1-receptor signalling is an important risk factor for AD^41^. We also identified four regulated proteins (dystrophin, glial fibrillary acidic protein [GFAP], myelin basic protein [MBP], and antigen KI-67 [MKI67]), which were highly expressed in AD tissue and were associated with IGF1 upstream regulation. This IGF1-associated signalling interaction could contribute to the pathogenesis of AD. Another growth factor protein identified was BDNF, which is associated with energy homeostasis^42^ and plays a neuroprotective role in AD 43,44. Furthermore, decreased BDNF mRNA and protein levels are observed in tauopathies and neurodegenerative disease^45^. Interestingly, our results suggest that Myc may inhibit the activation of BDNF-associated upstream regulators (Fig. 6A). The Myc oncogene family of proteins are upregulated^46^ and induce apoptosis^47^ in AD. We found that seven downstream proteins (heat shock protein HSP 90-alpha [HSP90AA1], [MBP], [MKI67], phosphoglycerate mutase 1 [PGAM1], phosphoglycerate kinase 1 [PGK1], pyruvate kinase PKM, and ADP/ATP translocase 2 [SLC25A5]) were upregulated in AD tissue and were upstream regulators of Myc. According to our data, activation of Myc may inhibit BDNF, which is known to play a neuroprotective role in AD. Tyrosine-protein kinase ZAP-70 (ZAP70; SRK), which regulates the adaptive immune response, was one of the kinase proteins identified. Here, we identified four downstream proteins (HSP90AA1, ITPR1, E3 ubiquitin-protein ligase MYCBP2, and plectin [PLEC]) associated with the upstream regulator ZAP70. ITPR1 is particularly important for the activation of apoptosis pathways downstream from the ER via activation of various kinases^48^,^49^,^50^ (Fig. 6A). These data suggest that activated ZAP70 may induce neuronal apoptosis through calcium-induced T lymphocyte apoptosis and/or immune response pathways. We also identified an inhibitor of upstream regulation, cyclosporin A (CsA), which is a biologic drug. CsA is an immunosuppressive cyclic peptide that binds cyclophilin A^51^. In addition, CsA prevents neuronal death^52^,^53^ and β-amyloid-induced late-phase long-term potentiation deficits^54^. These data suggest that CsA may be a potential therapeutic agent for the treatment of AD by negatively regulating downstream proteins (GFAP, HSP90AA1, ITPR1, and MDH2). A number of proteins were also associated with neurological disease pathways (Fig. 6B).

When dealing with human tissues, many variables must be taken into account that could potentially influence study results including age, PMI, sex, and other factors. The post-mortem integrity of post-translational protein modifications is largely unknown; however, in an AD patient population, factors such as age, gender, and PMI, had no significant effect on neuronal nuclear diameter^55^. Additionally, rapid autopsy programs are not well established^56^,^57^. Even with these variables, human brain-based research is still required and will aid our understanding of disease pathology^58^. In particular, when considering recent findings related to pathological analyses of the CA4 and DG fields from patients with AD. Notably, our group was the first to provide a molecular study of the CA4 and DG subfields in AD^12^.

AD is classically diagnosed by neuropsychological testing combined with neuroimaging. Both methods are non-invasive; however, both approaches are also not very sensitive. One recent addition to the approaches adopted to clinically diagnose this debilitating disease is the use of biomarkers, including β-amyloid and tau, measured in the cerebral spinal fluid^59^,^60^. More specificity was introduced with the use of PET and Pittsburgh compound B, which can identify amyloid deposits. With our approach, disease diagnosis can be further improved, since proteomic changes can now be observed at high resolution on a cellular level. Obviously, our method has the disadvantage of being invasive and can only be performed post-mortem, limiting most of our observations to the late stages of AD; however, we believe that this proteomic approach will be particularly useful for examining connectomics in AD in which amyloid-induced neurotoxicity induces a loss of connections between neurons, thereby initiating the neurodegenerative process. Consequently, the five putative AD biomarkers (MDH2, PCLO, TRRAP, YWHAZ, and MUC19) are not just potential biomarkers. Instead, together with the five upstream signalling factors (IGF1, BDNF, ZAP70, MYC, and cyclosporin A) they could help uncover the molecular mechanisms mediating AD or different neurodegenerative diseases.

## Methods

### Ethics statement and collection of human tissues

All experiments were approved by the local Ethic Committee in accordance with the Spanish and European legislation on this topic. Methods of collection for human brain tissues were performed in accordance with procedures that were approved by the Ethics Committee of the University of Barcelona.

### Chemicals

All chemicals were of the highest purity obtainable. Water, formic acid, trifluoroacetic acid (TFA), acetonitrile (ACN), and methanol were purchased from Biosolve B. V. (Valkenswaard, the Netherlands). Ammonium bicarbonate, ethanol (EtOH), α-cyano-4-hydroxycinnamic acid, aniline (ANI), sinapinic acid (SA), and 1,1,1,3,3,3-hexafluoro-2-propanol reagents for Toluidine blue staining were purchased from Sigma-Aldrich (Saint-Quentin Fallavier, France). Trypsin was purchased from Promega (Charbonnieres, France).

### Human tissue

Control and AD hippocampal specimens were acquired from the Barcelona Brain Bank in Spain. Control hippocampi were obtained from donors who had no known history of neurological or psychiatric disease. The inclusion criterion for AD tissue was a clinical diagnosis (AD-IV, AD-V). Once the tissue was received, it was immediately frozen and stored at –80 °C until further use. For this study, we used the following brain specimens: controls (age 55, 52, 63, 68, and 69 years with a post-mortem interval (PMI) of 9, 3, 6, 4, and 3 h, respectively), AD samples (age 79, 83, 75, 77, and 56 years, with a PMI of 18, 15, 11, 20, and 7 h, respectively) (Supplementary Information 1). Tissues were acquired and stored by the Barcelona Brain Bank, who also obtained informed consent and ethics committee approval.

### Tissue sample preparation

Ten-micrometre sections were obtained using a cryostat CM1510S (Leica Microsystems, Nanterre, France) and mounted onto indium-tin oxide (ITO)-coated conductive glass slides (Bruker Daltonics, Bremen, Germany). Sections were then vacuum-dried in a desiccator for 10 min and placed in a series of 70% EtOH, 95% EtOH, and chloroform solutions for 30 s each with concomitant drying under vacuum for 5 min. Sections were then dried and stored under vacuum. After MALDI analysis, the matrix was removed from some sections by washing slides with 70% EtOH; these sections were then stained using toluidine blue.

### Matrix deposition

For matrix deposition, an ionic matrix solution containing 10 mg/mL of SA and one equivalent of ANI in ACN/0.1% TFA (7:3, v/v) was prepared. The matrix was deposited on human hippocampal tissue sections with an automatic ImagePrep sprayer (Bruker Daltonics, Bremen, Germany), which creates an aerosol by vibrational vaporization and provides high-resolution images. SA/ANI deposition was performed using a custom method composed of an initialization phase to deposit the first layer of matrix on the slide followed by phases enabling favourable co-crystallization of matrix crystals and analytes (by virtue of alternating cycles of partial and complete drying).

### MALDI-TOF MSI

The MALDI matrix was applied using an ImagePrep device as described above. Protein imaging was then performed on an Ultraflex II MALDI-TOF/TOF instrument (Bruker Daltonics, Bremen, Germany) equipped with a smartbeam laser (Nd:YAG, 355 nm). Protein mass spectra were acquired in linear positive ion mode within a mass range of m/z 3,000–30,000. The distance between raster points was set to 80 μm with a total of 500 laser shots accumulated at a 200-Hz repetition rate for each pixel. Spectra were processed by baseline correction and smoothed using FlexAnalysis 3.2 software (Bruker Daltonics, Bremen, Germany). Image analysis and data visualization were performed with FlexImaging 2.1 software (Bruker Daltonics, Bremen, Germany). For statistical analysis, datasets obtained from FlexImaging were loaded into ClinProTools 2.2 software (Bruker Daltonics, Bremen, Germany) to conduct hierarchical clustering. Unsupervised clustering was selected with Euclidean as the distance method and Ward as the linkage method.

### MALDI image normalization

Normalization of MALDI imaging data is not appropriate for single spectra, but instead requires the dataset as a whole. Here, we normalized data with the aim of equalizing the total ion count for all spectra to counter the effect of image ‘hot spots’. One example of a hot spot occurring is when a large matrix crystal had been hit directly by the laser beam, resulting in higher spectrum intensity at this point. However, normalization has the disadvantage of artificially increasing the intensity of pure noise spectra. It is therefore possible to exclude noise spectra by defining a Y*mean*/Y*max* cutoff. Normalization was performed according to the following calculation:


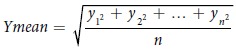


y = y value at a point in the spectrumn = number of data points in the spectrumIf Y*mean* < threshold factor × maximum value of the y-axis, then the spectrum will be normalized.

### Histological staining

For toluidine blue staining, tissue sections were removed from the SA/ANI matrix with 70% EtOH and dried; thereafter, sections were stained with toluidine blue for 1–2 min and washed in distilled water. After dehydration in a series of graded EtOH solutions, sections were placed in xylene, mounted, and examined by light microscopy^61^.

### Micro tissue extraction

Human brain tissue sections (20-μm thickness) were obtained using a cryostat CM1510S (Leica Microsystems, Nanterre, France) and mounted onto parafilm-covered slides. Micro-extraction was performed using microscopy to extract the CA4 and DG fields from AD and control hippocampal tissues.

### NanoLC-ESI ion-trap MS and MS/MS

After micro tissue extraction, analyses were performed on an ion-trap mass spectrometer (Esquire 3000 plus, Bucker Daltonics) equipped with a ESI ion source and on-line coupled to a nanoLC system. An injection of 0.5 μL of the digest was performed with a Switchos Autosampler (Dionex Corporation) and separation was performed on a C18 silica-bonded stationary phase (75 μm internal diameter, 150 mm long, 3 μm particle size, 100 Å pore size, Dionex). Samples were washed with 100% mobile phase A (95% water, 5% ACN, 0.1% formic acid) for 2 minutes at a flow rate of 10 μL/min. Peptides were then eluted using a linear gradient of 1%/minute mobile phase B (80% ACN, 20% water, 0.08% formic acid) for 70 minutes at a flow rate of 0.2 μL/min. The Esquire system was operated in a data-dependent MS/MS mode in which 1 MS full scan was followed by 1 MS/MS scan on the most-abundant peptide ion. Collision energy was set to 35%. Heated capillary temperature and electrospray voltage were 160 °C and 1.5 kV respectively^62^.

### Protein identification using label-free quantification and statistical analysis

Raw MS data from patients with AD and controls were processed for protein identification using Mascot Daemon (v.2.3.2) with a peptide mass tolerance of 100 ppm and a fragment mass tolerance of ±0.8 Da at a false discovery rate (FDR) of 1% for both proteins and peptides. All peak lists were identified with the human International Protein Index (IPI) database (v3.87; containing 91,491 entries) with a decoy using the following parameters: enzyme: trypsin, missed cleavages: ≤2, fixed modification: carbamidomethyl (C), variable modifications: oxidation (M), and phosphorylation (S, T, Y). For relative protein quantification, the Mascot output file (csv format) was first imported into Microsoft Excel with additional filtering applied as follows: protein score ≥40, elimination of contaminants, and reversed sequences for each IPI name. A spectral index (SI) was calculated by summing all spectral counts using only unique peptides. Second, spectral indices from respective IPI names were normalized to the number of total counts estimating the relative amount of varying IPI names within a relative sample. Third, the average ratio of normalized SI was calculated between control tissue and AD tissue. Finally, a Student’s t-test was performed for each IPI name (p <0.05). To control the FDR, the resulting p values were adjusted for multiple testing according to the method of Benjamini and Hochberg^63^ and compared with a significance level of alpha = 0.05. A protein was accepted as differentially expressed if it exhibited a ratio >1.8 AD or control tissue and an adjusted p value <0.05.

### Gene annotation and upstream regulator analysis

Gene ontology annotation enrichment analysis was performed using the DAVID Bioinformatics Resource (v6.7) developed by NIAID, at the National Institutes of Health. Ingenuity Pathway Analysis (IPA; Ingenuity Systems, http://www.ingenuity.com) was used to functionally annotate genes implicated in disease and search for common upstream regulators.

### Western blotting

For western blot analyses, protein extracts containing either 15 μg of AD or control lysate. Blots were then incubated overnight with primary antibodies [rabbit polyclonal anti-TRRAP (1:1000, Abcam), rabbit polyclonal anti-YWHAZ (1:1000, AbFrontier), mouse monoclonal anti-PCLO (1:1000, Abcam), mouse monoclonal anti-MDH2 (1:1000, Abcam), and mouse monoclonal anti-β-actin (1:5000, Sigma)] diluted in 5% skimmed milk powder in Tris-buffered saline (TBS) at 4 °C. Primary antibodies were incubated with peroxidase-conjugated anti-rabbit IgG (1:5000, Sigma) or peroxidase-conjugated anti-mouse I gG (1:5000, Sigma) secondary antibodies at RT. Antibody-reactive protein bands were visualized with an ECL detection reagent (GE Healthcare).

### Optimization for peptide synthesis and SRM analysis in the MS instrument

To perform SRM measurements, isotopically-labelled peptides were optimized with Optimizer software (MassHunter optimizer, version B. 0.3, Agilent, Palo Alto, CA, USA) to obtain the best analysis conditions for the triple quadrupole MS instrument. The optimization process selected fragment moieties from precursor ions and set the collision energy of fragment ions in each peptide. These samples were analysed with a nanoLC chip SRM/triple quadrupole MS, coupled with a 1200 Nano HPLC-chip microfluidic device (Agilent Technologies, Santa Clara, CA, USA). Samples (6 μL; 1.5 μg of protein) were injected via an autosampler and loaded onto the chip enrichment column, trapping 160 nL with a capillary pump, with a loading flow of 4 μL/min using a mobile phase composed of 0.1% formic acid in water. A separation column (Zorbax C18, 75 μm × 150 mm, 5 μm) was used for peptide separation, setting the analytical flow rate to 400 nL/min. Mobile phase A consisted of 97% water/3% ACN/0.1% formic acid, and mobile phase B consisted of 90% ACN/10% water/0.1% formic acid. The capillary pump flow rate was set at 4 μL/min for loading and washing the samples, using a 100% water/0.1% formic acid mobile phase. The LC gradient used increasing concentrations of B with the total run time equalling 60 min. ESI was accomplished by applying a spray voltage of 1840 V. MS instrument settings included a drying gas flow rate of 2.5 L/min, with a gas temperature of 325 °C, and an applied fragmentor voltage of 135 V(Supplementary Information 3).

## Additional Information

**How to cite this article**: Ho Kim, J. *et al*. Proteome-wide characterization of signalling interactions in the hippocampal CA4/DG subfield of patients with Alzheimer's disease. *Sci. Rep*. **5**, 11138; doi: 10.1038/srep11138 (2015).

## Supplementary Material

Supplementary Information

## Figures and Tables

**Table 1:** Upregulation and downregulation proteins list (Ratio ≥ 1.80 or p-value < 0.05*), SI = spectral index Asterisks indicate proteins with ratios that were significantly different from 1 (one-sample t-test, p < 0.05*).

**IPI**	**GENE SYMBOL**	**Sequencecoverage (%)**	**No ofpeptide**	**AD(SI)**	**WT(SI)**	**AverageFold Change (AD/WT)**	**Adjustedp-value**	**IPI**	**GENESYMBOL**	**Sequencecoverage (%)**	**No ofpeptide**	**AD(SI)**	**WT(SI)**	**AverageFold Change (AD/WT)**	**Adjustedp-value**
**Upregulaton proteins**															
IPI00470912	C2orf16*	9.0	12	7.53	0.00	n.a	0.00982	IPI01018212	PKM2	22.2	34	6.87	2.92	2.35	0.01708
IPI00216864	PDZRN4*	9.5	16	5.28	0.00	n.a	0.00731	IPI00333696	HEATR5B	7.8	21	5.72	2.43	2.35	0.01892
IPI00876841	ZSWIM8 *	4.6	9	4.23	0.25	16.72	0.01489	IPI00069084	TRRAP	2.3	18	3.73	1.64	2.28	0.01892
IPI00219446	PEBP1*	28.6	10	3.30	0.24	13.96	0.00040	IPI00943735	ANKRD36	6.3	10	3.15	1.38	2.28	0.01892
IPI00871535	SPTAN1	3.0	13	9.35	1.01	9.24	0.01892	IPI00642259	DST	2.1	29	12.91	5.72	2.26	0.01899
IPI00964552	COL12A1*	5.4	28	6.88	0.97	7.08	0.00444	IPI00218474	ENO3	14.7	14	2.17	0.98	2.21	0.01892
IPI00084828	STXBP1*	11.8	9	3.78	0.57	6.67	0.01588	IPI00549725	PGAM1	17.5	9	3.78	1.74	2.17	0.01892
IPI00413144	C15orf42	6.2	10	8.22	1.27	6.48	0.01766	IPI00291006	MDH2	27.9	25	11.18	5.21	2.15	0.01892
IPI01014674	MUC19*	31.6	11	10.22	1.89	5.41	0.01349	IPI00414123	CRMP1	10.9	6	0.99	0.46	2.14	0.02350
IPI00954159	AHCTF1P1	6.0	20	8.06	1.52	5.31	0.01856	IPI00025363	GFAP	61.1	195	38.93	18.50	2.10	0.01856
IPI00382470	HSP90AA1	7.2	8	5.18	1.01	5.11	0.01892	IPI00550232	XIRP2	3.6	18	6.33	3.09	2.05	0.01892
IPI00169383	PGK1	14.9	4	3.22	0.67	4.79	0.01839	IPI00045512	HMCN1	3.2	30	6.90	3.39	2.04	0.01892
IPI00879193	TTC28	5.1	13	6.89	1.46	4.73	0.01856	IPI00006091	DMD	3.3	9	5.55	2.73	2.03	0.01899
IPI00007188	SLC25A5	18.6	5	1.00	0.22	4.57	0.01856	IPI00215715	CAMK2A	13.5	14	4.42	2.18	2.03	0.01892
IPI00884904	AHNAK2	3.4	15	10.25	2.79	3.67	0.01892	IPI00930130	TUBB6	48.0	107	1.92	0.95	2.02	0.01892
IPI01011955	MKI67	7.2	21	5.33	1.53	3.49	0.01892	IPI00419908	GPR179	4.9	9	4.24	2.10	2.02	0.02350
IPI00218658	ITPR1	5.8	13	4.20	1.27	3.31	0.01856	IPI00171636	NAV1	5.8	10	3.76	1.92	1.95	0.01892
IPI00328089	HIVEP3*	6.4	17	8.16	2.53	3.22	0.01203	IPI01015973	MUC16	8.6	233	7.47	3.87	1.93	0.01892
IPI00413173	MKI67	3.4	10	10.05	3.52	2.85	0.01856	IPI00004233	MKI67	7.2	21	9.39	4.93	1.90	0.02167
IPI00411656	PCLO*	3.2	26	16.37	5.81	2.82	0.01588	IPI00925013	MLL3*	12.5	26	8.01	4.25	1.89	0.01689
IPI00740909	ALMS1	4.5	21	6.37	2.28	2.80	0.01856	IPI00218621	SON	7.6	98	7.24	3.86	1.87	0.02078
IPI00398002	PLEC	4.1	28	7.85	3.00	2.61	0.01892	IPI00329573	COL12A1	7.2	30	5.12	2.79	1.84	0.01932
IPI00789624	PCLO	6.7	40	13.14	5.08	2.59	0.01839	IPI00797574	BOD1L	5.9	23	11.54	6.38	1.81	0.01856
IPI00749231	GPR98	3.5	17	3.19	1.24	2.58	0.01892	IPI00021907	MBP	32.2	41	5.19	2.88	1.80	0.02579
IPI00289776	MYCBP2	4.2	18	13.60	5.49	2.48	0.01892								
IPI00169307	ARHGAP21*	6.3	27	7.77	3.17	2.45	0.01489								
IPI00854820	PPRC1	6.6	14	2.86	1.18	2.42	0.01892								
IPI00215631	VCAN	4.0	21	4.29	1.79	2.39	0.01892								
IPI00102752	RBM15	7.6	20	4.99	8.89	0.56	0.01892	IPI00788168	MAST4	3.5	8	1.23	3.12	0.39	0.01892
IPI00014845	DNAH8	3.9	29	2.63	4.73	0.56	0.01892	IPI00908811	CKB	32.3	26	1.05	2.69	0.39	0.01892
IPI00217051	NAV3	11.7	33	8.65	15.58	0.56	0.01892	IPI00006451	NSF	5.0	4	1.01	2.58	0.39	0.01892
IPI00044608	PLIN4	17.2	30	7.38	13.38	0.55	0.01892	IPI00031411	FAT1	3.0	17	2.66	7.08	0.38	0.01892
IPI00854629	OBSCN	2.3	33	3.83	6.97	0.55	0.01892	IPI00983524	MBP	58.6	59	0.62	1.76	0.35	0.01856
IPI00221325	RANBP2	5.3	14	3.98	7.24	0.55	0.02154	IPI00445401	HUWE1*	5.6	27	2.39	6.99	0.34	0.01203
IPI00868928	KIF26B	5.5	19	3.45	6.39	0.54	0.01892	IPI00257508	DPYSL2*	23.3	27	3.22	10.01	0.32	0.01203
IPI01018628	UTY	11.6	24	5.10	9.47	0.54	0.01892	IPI00021263	YWHAZ*	17.0	12	0.84	2.65	0.32	0.01489
IPI00018352	UCHL1	19.2	10	1.77	3.30	0.53	0.01892	IPI00479743	POTEE	14.1	40	0.82	2.75	0.30	0.01892
IPI00815697	NAV2	12.0	31	2.12	4.00	0.53	0.02549	IPI01019113	TUBB	49.6	155	0.23	0.79	0.30	0.01756
IPI00003935	HIST2H2BE	19.0	9	1.27	2.39	0.53	0.01892	IPI00006510	TUBB1	11.7	11	0.29	0.99	0.30	0.01892
IPI00008161	ATP12A	5.9	12	0.77	1.46	0.53	0.01892	IPI01019105	UTY	4.7	10	1.29	4.57	0.28	0.01892
IPI00018511	TUBB4Q	11.7	12	0.51	0.98	0.52	0.01977	IPI00478021	DNAH8*	4.0	29	2.06	7.68	0.27	0.01676
IPI00952742	SHANK2	4.6	14	2.05	4.00	0.51	0.01892	IPI00217002	KIAA2018	7.2	11	1.48	5.65	0.26	0.01856
IPI00297593	USP34	4.0	25	3.75	7.30	0.51	0.01892	IPI00853390	GPR112*	5.8	34	1.84	7.91	0.23	0.00982
IPI00306457	FNDC1	9.4	10	2.74	5.42	0.51	0.01892	IPI00443415	MCC*	5.5	9	0.99	4.32	0.23	0.01203
IPI00019848	HCFC1	7.5	15	3.51	6.95	0.51	0.02078	IPI00956686	ATXN7*	15.6	11	0.88	4.54	0.19	0.01203
IPI00914628	USP24	4.5	9	1.76	3.48	0.50	0.02135	IPI00157790	KIAA0368	4.2	7	0.99	5.17	0.19	0.01856
IPI00026320	UBR5	5.1	15	6.07	12.42	0.49	0.01892	IPI00220717	RBM15*	7.6	20	1.03	5.64	0.18	0.00731
IPI00215877	APC	7.6	41	3.85	7.89	0.49	0.01892	IPI00795015	SACS*	1.9	15	0.99	5.78	0.17	0.00731
IPI00946001	ONECUT2	26.8	15	3.48	7.17	0.49	0.01891	IPI00738216	KIAA0947*	7.1	15	0.62	3.82	0.16	0.01203
IPI00298994	TLN1	6.9	18	4.76	9.93	0.48	0.01856	IPI00642705	ARID1A*	7.7	25	1.33	8.27	0.16	0.00731
IPI00914890	MAST4	3.3	8	1.17	2.47	0.47	0.02154	IPI01014270	APOB	4.6	21	1.33	9.55	0.14	0.01856
IPI00788188	DYNC2H1	5.4	24	2.83	6.05	0.47	0.01892	IPI00740545	POTEI*	11.2	26	0.21	1.54	0.14	0.00507
IPI00007750	TUBA4A	37.4	56	0.55	1.19	0.46	0.01856	IPI00790327	ATP1A3*	8.7	27	0.40	4.10	0.10	0.01489
IPI00026272	HIST1H2AE	21.8	6	0.76	1.67	0.46	0.01892	IPI00879810	SPTAN1	5.3	18	1.00	10.50	0.10	0.01892
IPI00985241	MBP	67.5	59	1.00	2.21	0.45	0.01892	IPI00917728	NEB*	3.7	58	0.80	9.19	0.09	0.00549
IPI00215628	VCAN	5.5	9	1.17	2.75	0.43	0.02109	IPI00871227	HMCN1*	3.1	15	0.29	4.56	0.06	0.01203
IPI00643465	TACC2	3.2	7	1.17	2.76	0.42	0.01892	IPI00386035	FLG*	11.7	21	0.00	6.61	n.a	0.00731
IPI00179298	HUWE1*	5.6	43	4.55	10.74	0.42	0.01708	IPI00012645	SPTBN2*	5.9	10	0.00	6.66	n.a	0.00741
IPI00847978	FAT3	1.0	13	2.56	6.06	0.42	0.02078								

**Table 2:** Annotated upstream regulators listed according to z-scores and p values.

**Upstream Regulator**	**Molecule Type**	**Activation z-score**	**p-value**	**Target molecules in regulated proteins**
ZAP70	Kinase	2.000	4.12E–06	HSP90AA1, ITPR1, MYCBP2, PLEC
IGF1	Growth factor	1.970	6.92E–03	DMD,GFAP, MBP, MKI67
BDNF	Growth factor	1.953	8.33E–04	GFAP, HSP90AA1, ITPR1, MBP
MYC	Transcription regulator	1.865	2.49E–03	HSP90AA1, MBP, MKI67, PGAM1, PGK1, PKM, SLC25A5
Cyclosporin A	Biologic drug	−1.982	7.61E–03	GFAP, HSP90AA1, ITPR1, MDH2
