# Supplementary material for: Proteome-wide characterization of signalling interactions in the hippocampal CA4/DG subfield of patients with Alzheimer’s disease
Source: Sci Rep. 2015 Jun 10;5:11138. doi: 10.1038/srep11138 (PMC4462342; doi:10.1038/srep11138)

Proteome-wide characterization of signalling interactions in the hippocampal CA4/DG subfield of patients with Alzheimer's disease

Jae Ho Kim[1,2¶], Julien Franck[3,¶], Taewook Kang[1,2,4¶], Helmut Heinsen[5], Rivka Ravid[6], Isidro Ferrer[7], Mi Hee Cheon[2,4], Joo-Yong Lee[4], Jong Shin Yoo[2,4], Harry W Steinbusch[8], Michel Salzet[3,], Isabelle Fournier[3,*] and Young Mok Park[1,2,4*]

[1]Center for Cognition and Sociality, Institute for Basic Science, Daejeon 305-811, Republic of Korea

[2]Mass Spectrometry Research Center, Korea Basic Science Institute, 804-1 Yangcheong-ri, Ochang-eup, Cheongwon-gun, Chungbuk 363-883, Republic of Korea

[3]Inserm U-1192, Laboratoire de Protéomique, Réponse Inflammatoire, Spectrométrie de Masse (PRISM), Université de Lille 1, Cité Scientifique, 59655 Villeneuve D'Ascq, France

[4]Graduate School of Analytical Science and Technology, Chungnam National University, Daejeon 305-764, Republic of Korea

[5]Department of Psychiatry, Morphological Brain Research Unit, University of Würzburg, Würzburg, Germany

[6]Brain Bank Consultants, Amsterdam, The Netherlands

[7]Institut de Neuropatologia, Servei Anatomia Patologica, IDIBELL-Hospital Universitari de Bellvitge, Universitat de Barcelona, Spain

[8]School for Mental Health and Neuroscience, Department of Translational Neuroscience, Maastricht University, Maastricht, The Netherlands

¶Co-first authors

***Corresponding authors**

Supplementary Information 1. Demographic of Alzheimer's disease and control subjects
Supplementary Information 2. Dataset of MUC19 isoform 5
Supplementary Information 3. SRM dataset of MDH2 protein from AD and control tissues.

Supplementary Information 1. Demographic of Alzheimer's disease and control subjects

Group	Case label	Age of death (years old)	Gender	Post-mortem time (hour)	Diagnosis	Hippocampus	Width (cm)	Length (cm)
Control	1	55	Male	9	Unaffected	Yes	1.3	1.0
	2	52	Male	3	Unaffected	Yes	0.5	1.4
	3	63	Male	6	Unaffected	Yes	1.2	1.4
	4	68	Female	4	Unaffected	Yes	1.2	1.8
	5	69	Male	3	Unaffected	Yes	1.0	1.8
AD	6	79	Male	18	AD Ⅳ	Yes	1.1	1.5
	7	83	Female	15	AD Ⅳ	Yes	0.9	1.4
	8	75	Male	11	AD Ⅴ	Yes	1.1	1.5
	9	77	Male	20	AD Ⅳ	Yes	0.8	1.8
	10	56	Female	7	AD Ⅳ	Yes	1.0	1.9

Mean age of death (Mean ± S.E.M.): control cases, 61.4 ± 3.4 yr; AD cases, 74 ± 4.7 yr.
Mean post-mortem intervals (mean ± S.E.M.): control cases, 5 ± 1.1 hrs; AD cases, 14.2 ± 2.4 hrs

Supplemental_Material_2

Domains within *Homo sapiens* Mucin-19 Protein

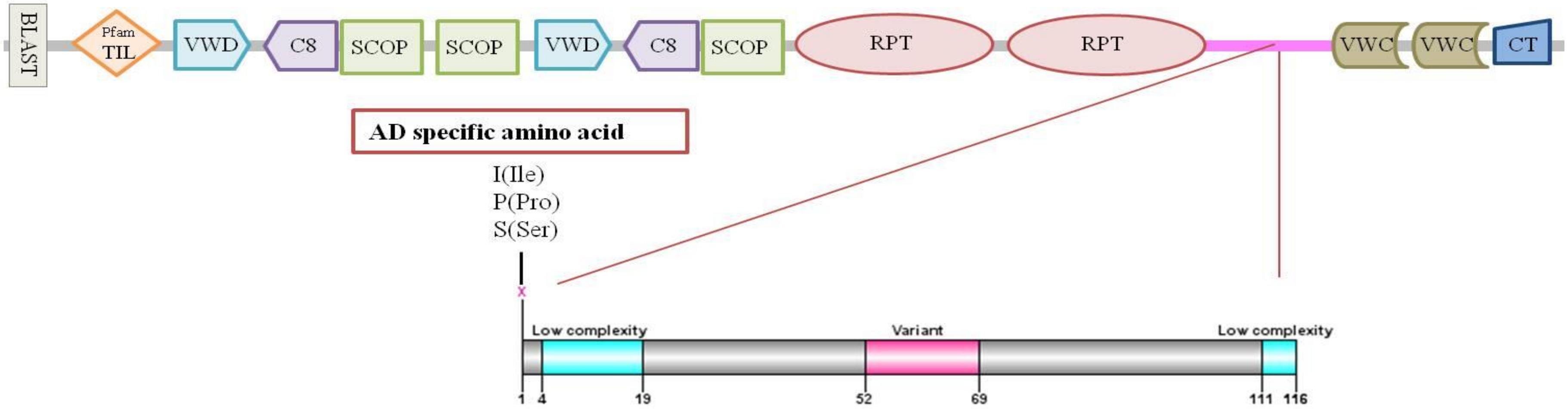

Protein alignment

|H0YH61|Muc-19_HUMAN: 0 ---**X**TSTDVGVATGVGMATGITN 20
|Q7Z5P9|Muc-19_HUMAN: 5828 NTEATTSLGNGGTTEAGSKIVTTGITTGTTIVPGSFNTKATTSTDVGVATGVGMATGITN 5887

|H0YH61|Muc-19_HUMAN: 21 IISGRSQPTGSKTGYTVTGSGTTALPGGFRT**EATTFKGDVGTTEAEISS**GNTPGSTGVTS 80
|Q7Z5P9|Muc-19_HUMAN: 5888 IISGRSQPTGSKTGYTVTGSGTTALPGGFRT---------------------------GNTPGSTGVTS 5929

|H0YH61|Muc-19_HUMAN: 81 SQEGTTVVSSGITGIPETSISGPSKEASDKTTAPGP---------------------------------- 116
|Q7Z5P9|Muc-19_HUMAN: 5930 SQEGTTVVSSGITGIPETSISGPSKEASDKTTAPGPPTTVTASTGVKETSETGVQTGSTL 5989

Supplementary Information 3. SRM dataset of MDH2 protein from AD and control tissues

AD vs Control Brain Sample

– Endogenous Light peak / Heavy standard peak Ratio

MDH2		AD			Control		
1.5ug/750f		Heavy Area	Light Area	L/H	Heavy Area	Light Area	L/H
fragment ion	1	103032	16872	0.164	96540	8396	0.087
	2	87124	14392	0.165	80981	6963	0.086
	3	24615	3730	0.152	22016	1861	0.085
	4	43530	6546	0.150	38557	3379	0.088
Fragment ion ratio	F2/F1	0.846	0.853		0.839	0.829	
	F3/F1	0.239	0.221		0.228	0.222	
	F4/F1	0.422	0.388		0.399	0.402	

MDH2		AD			Control		
1ug/500f		Heavy Area	Light Area	L/H	Heavy Area	Light Area	L/H
Fragment ion	1	56246	9244	0.164	58506	5405	0.092
	2	47313	8088	0.171	51799	5042	0.097
	3	13443	2230	0.166	13672	1356	0.099
	4	22842	4073	0.178	24466	2397	0.098
Fragment ion ratio	F2/F1	0.841	0.875		0.885	0.933	
	F3/F1	0.239	0.241		0.234	0.251	
	F4/F1	0.406	0.441		0.418	0.443	

AD vs Control Sample –Retention Time

MDH_2		AD-RT		Control-RT	
1.5ug/750f		Heavy	Light	Heavy	Light
Fragment ion	1	12.06	12.08	12.26	12.24
	2	12.08	12.10	12.25	12.28
	3	12.03	12.09	12.30	12.28
	4	12.05	12.03	12.25	12.3
RT average		12.06	12.08	12.27	12.28
RT STDEV		0.02	0.03	0.02	0.03
RT CV%		0.17	0.26	0.19	0.21

MDH_2		AD-RT		Control-RT	
1ug/500f		Heavy	Light	Heavy	Light
Fragment ion	1	12.35	12.43	12.39	12.42
	2	12.34	12.38	12.35	12.39
	3	12.36	12.40	12.39	12.43
	4	12.35	12.39	12.37	12.38
RT average		12.35	12.40	12.38	12.41
RT STDEV		0.01	0.02	0.02	0.02
RT CV%		0.07	0.17	0.15	0.19

MDH2 Chromatogram

- Fragment ion overlapping

AD 1.5ug

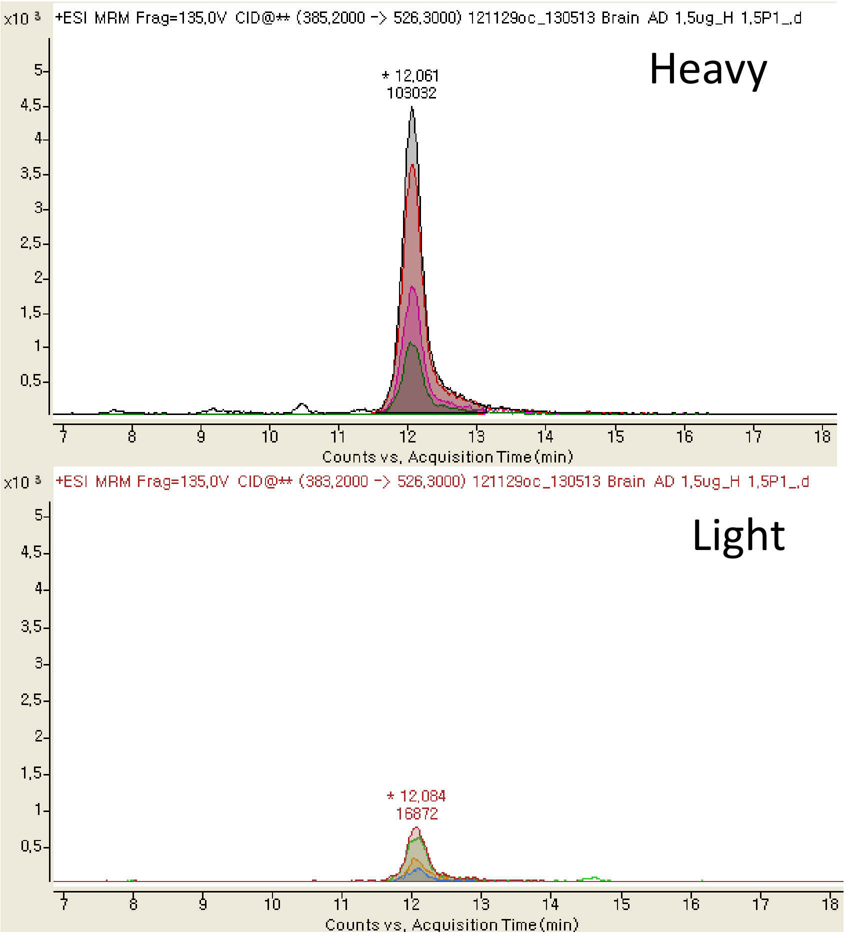

Control 1.5ug

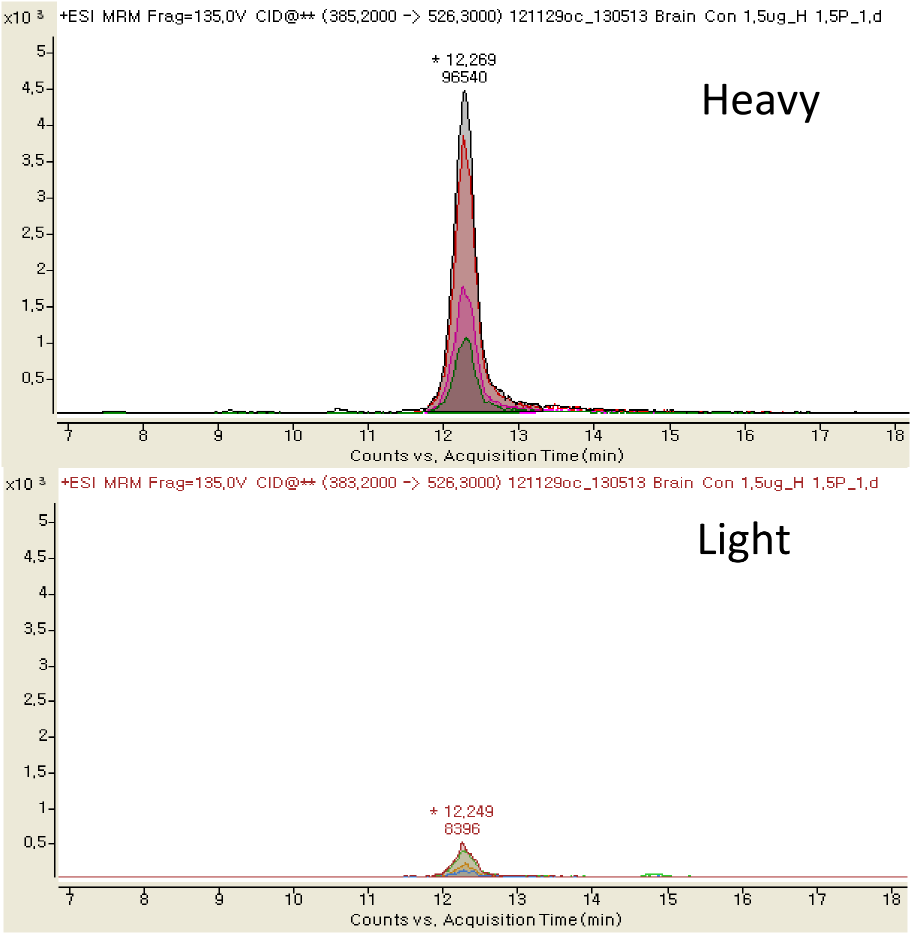

AD 1.5ug

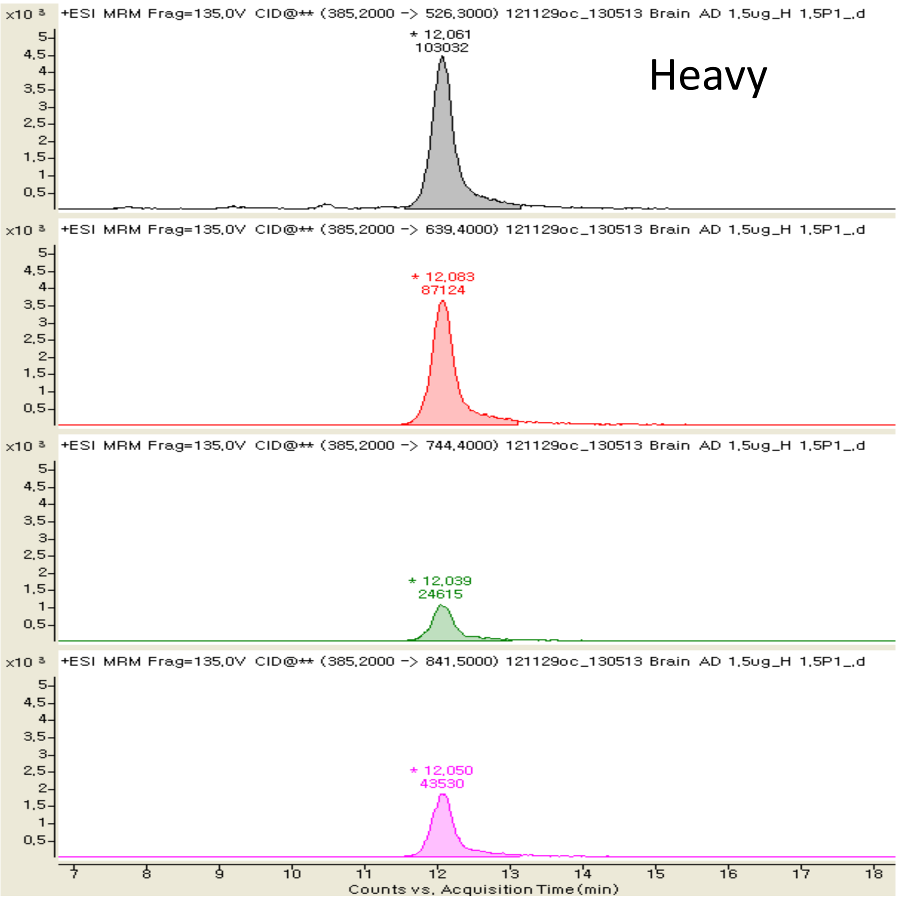

Control 1.5ug

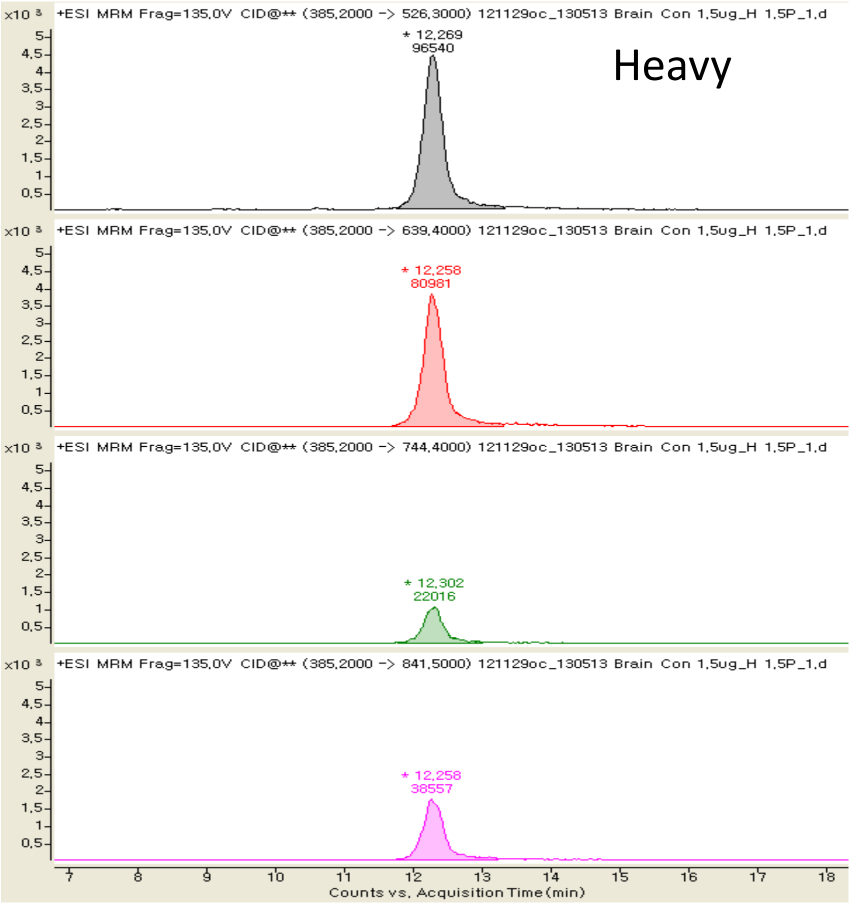

AD 1.5ug

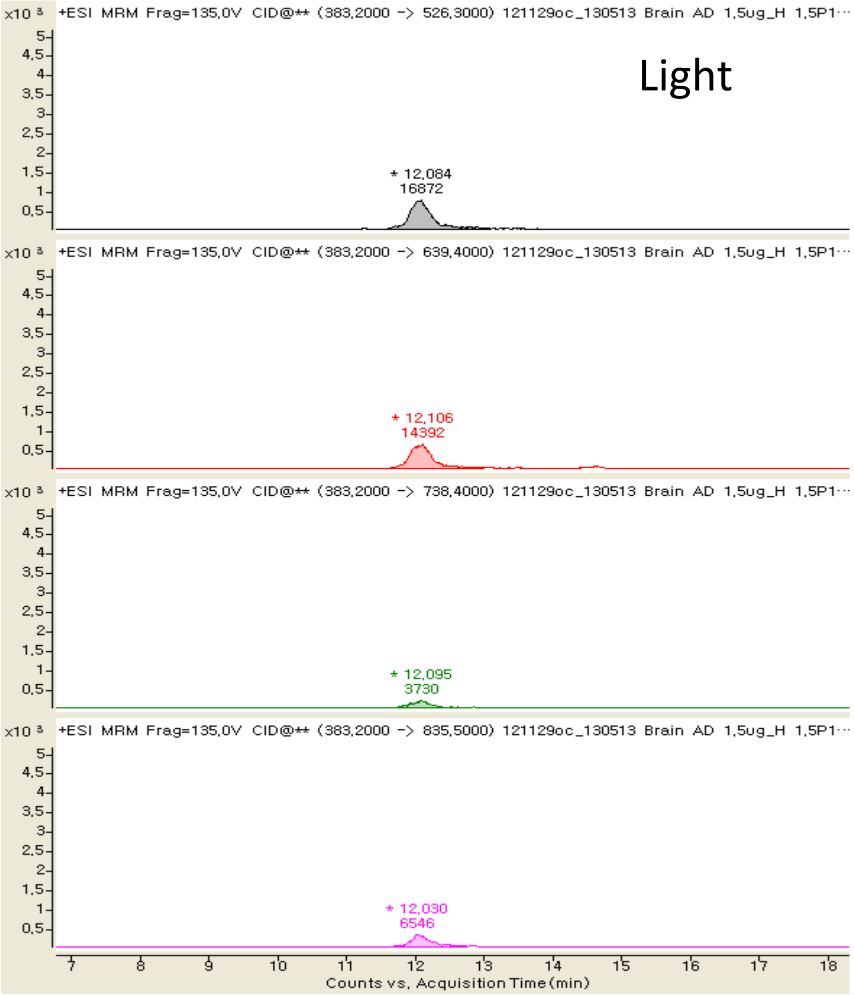

Control 1.5ug

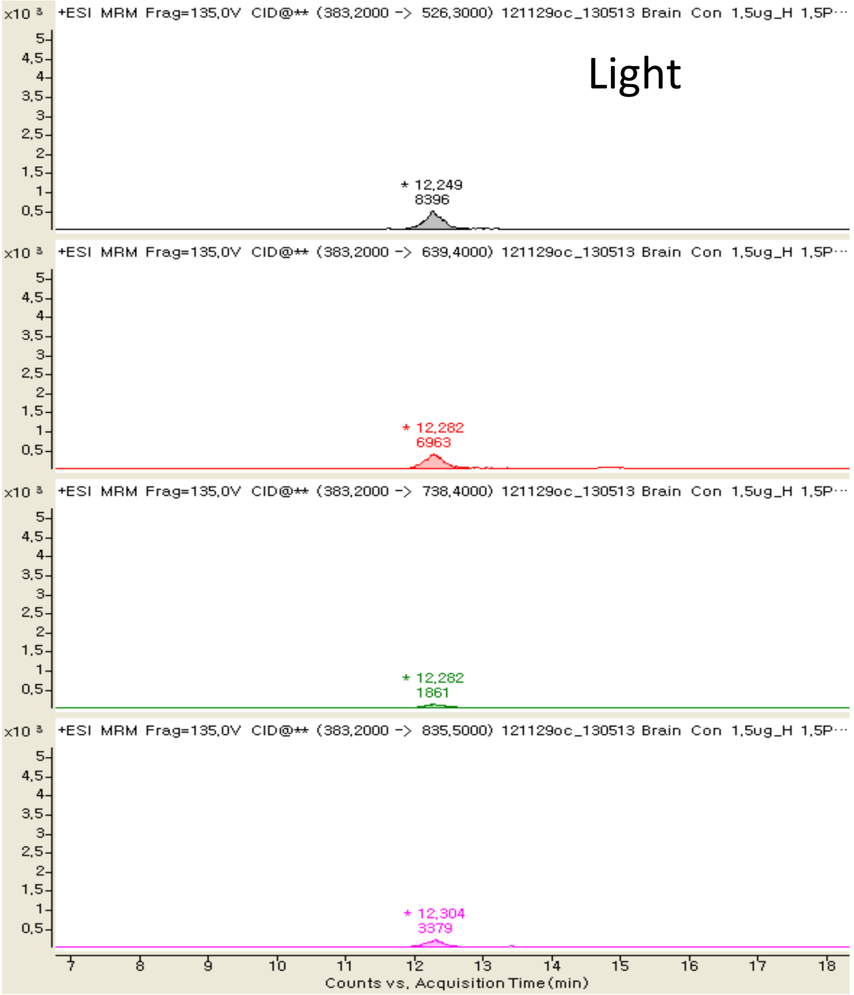

Supplement: Supplementary Information [file srep11138-s1.pdf]
